# N-of-1 health optimization: Digital monitoring of biomarker dynamics to gamify adherence to metabolic switching

**DOI:** 10.1093/pnasnexus/pgae214

**Published:** 2024-05-30

**Authors:** Peter Wang, Xavier Tadeo, Han Shi Jocelyn Chew, Yoann Sapanel, Yoong Hun Ong, Nicole Yong Ting Leung, Edward Kai-Hua Chow, Dean Ho

**Affiliations:** Department of Biomedical Engineering, College of Design and Engineering, National University of Singapore, Singapore 117583, Singapore; Institute for Digital Medicine (WisDM), Yong Loo Lin School of Medicine, National University of Singapore, Singapore 117456, Singapore; The N.1 Institute for Health (N.1), National University of Singapore, Singapore 117456, Singapore; Institute for Digital Medicine (WisDM), Yong Loo Lin School of Medicine, National University of Singapore, Singapore 117456, Singapore; The N.1 Institute for Health (N.1), National University of Singapore, Singapore 117456, Singapore; Alice Lee Centre for Nursing Studies, Yong Loo Lin School of Medicine, National University of Singapore, Singapore 117597, Singapore; Institute for Digital Medicine (WisDM), Yong Loo Lin School of Medicine, National University of Singapore, Singapore 117456, Singapore; Singapore's Health District @ Queenstown, Yong Loo Lin School of Medicine, National University of Singapore, Singapore 117456, Singapore; Institute for Digital Medicine (WisDM), Yong Loo Lin School of Medicine, National University of Singapore, Singapore 117456, Singapore; The N.1 Institute for Health (N.1), National University of Singapore, Singapore 117456, Singapore; Institute for Digital Medicine (WisDM), Yong Loo Lin School of Medicine, National University of Singapore, Singapore 117456, Singapore; The N.1 Institute for Health (N.1), National University of Singapore, Singapore 117456, Singapore; Department of Biomedical Engineering, College of Design and Engineering, National University of Singapore, Singapore 117583, Singapore; Institute for Digital Medicine (WisDM), Yong Loo Lin School of Medicine, National University of Singapore, Singapore 117456, Singapore; The N.1 Institute for Health (N.1), National University of Singapore, Singapore 117456, Singapore; Department of Pharmacology, Yong Loo Lin School of Medicine, National University of Singapore, Singapore 117600, Singapore; Cancer Science Institute of Singapore, National University of Singapore, Singapore 117599, Singapore; NUS Centre for Cancer Research (N2CR), Yong Loo Lin School of Medicine, National University of Singapore, Singapore 117599, Singapore; Department of Biomedical Engineering, College of Design and Engineering, National University of Singapore, Singapore 117583, Singapore; Institute for Digital Medicine (WisDM), Yong Loo Lin School of Medicine, National University of Singapore, Singapore 117456, Singapore; The N.1 Institute for Health (N.1), National University of Singapore, Singapore 117456, Singapore; Singapore's Health District @ Queenstown, Yong Loo Lin School of Medicine, National University of Singapore, Singapore 117456, Singapore; Department of Pharmacology, Yong Loo Lin School of Medicine, National University of Singapore, Singapore 117600, Singapore; The Bia-Echo Asia Centre for Reproductive Longevity and Equality, Yong Loo Lin School of Medicine, National University of Singapore, Singapore 117456, Singapore

**Keywords:** N-of-1 health, metabolic switching, biomarker, intermittent fasting, gamification

## Abstract

The digital health field is experiencing substantial growth due to its potential for sustained and longitudinal deployment. In turn, this may drive improved monitoring and intervention as catalysts for behavioral change compared to traditional point-of-care practices. In particular, the increase in incidence of population health challenges such as diabetes, heart disease, fatty liver disease, and other disorders coupled with rising healthcare costs have emphasized the importance of exploring technical, economics, and implementation considerations, among others in the decentralization of health and healthcare innovations. Both healthy individuals and patients stand to benefit from continued technical advances and studies in these domains. To address these points, this study reports a N-of-1 study comprised of sustained regimens of intermittent fasting, fitness (strength and cardiovascular training), and high protein, low carbohydrate diet and parallel monitoring. These regimens were paired with serial blood ketone, blood glucose (wearable and finger stick) and blood pressure readings, as well as body weight measurements using a collection of devices. Collectively this suite of platforms and approaches were used to monitor metabolic switching from glucose to ketones as energy sources—a process associated with potential cardio- and neuroprotective functions. In addition to longitudinal biomarker dynamics, this work discusses user perspectives on the potential role of harnessing digital devices to these dynamics as potential gamification factors, as well as considerations for the role of biomarker monitoring in health regimen development, user stratification, and potentially informing downstream population-scale studies to address metabolic disease, healthy aging and longevity, among other indications.

**Significance Statement**

Multiple digital devices were harnessed to monitor biomarker dynamics in response to a combination of intermittent fasting, fitness, and dietary interventions in an N-of-1 study. Metabolic switching, a process whereby a subject converts between sugar and fat as primary energy sources, was observed in subject N001's biomarker trajectories. More importantly, for N001, the maintenance of a dynamic biomarker trajectory served as a promoter of adherence to the regimens. On a generalizable level, this work illuminates the importance of data set collection that is longitudinal and captures the dynamics of user response to variable and combinatorial interventions. These findings reflect the potential of gamifying adherence to different regimens in order to maintain biomarker trajectories that may lead to improved health management.

## Introduction

The role of digital health towards enabling and supporting personalized, community and population health is being widely studied (1–3). For example, emerging platforms are investigating how to decentralize and democratize the monitoring and management of conditions such as heart disease, diabetes, frailty, and other conditions that are increasing in global incidence due to rapidly aging societies and factors which include but are not limited to cardiopulmonary disease and diabetes mellitus (4–7). Solutions being developed range from mobile applications to wearable devices, and the data obtained from these solutions are being applied to artificial intelligence (AI) and machine learning-based predictive modeling for clinical decision support and other uses (8, 9). Notably, multiple wearable devices have utilized sweat-based analysis to dynamically assess the physiological state (e.g. glucose, ketone, lactate, sodium/potassium ions) and the risk of metabolic syndrome of the subjects (10–13). Barriers to sustainably implementing digital health solutions have included waning user adherence towards wearables and mobile applications, challenges with integrating predictive modeling into real-world clinical workflows, inter-operability issues with electronic health records and other considerations (14–16). Collectively, these factors are important to consider as evidence generation will be essential towards consistently incorporating digital health into widespread health and medical use.

**Fig. 1.**
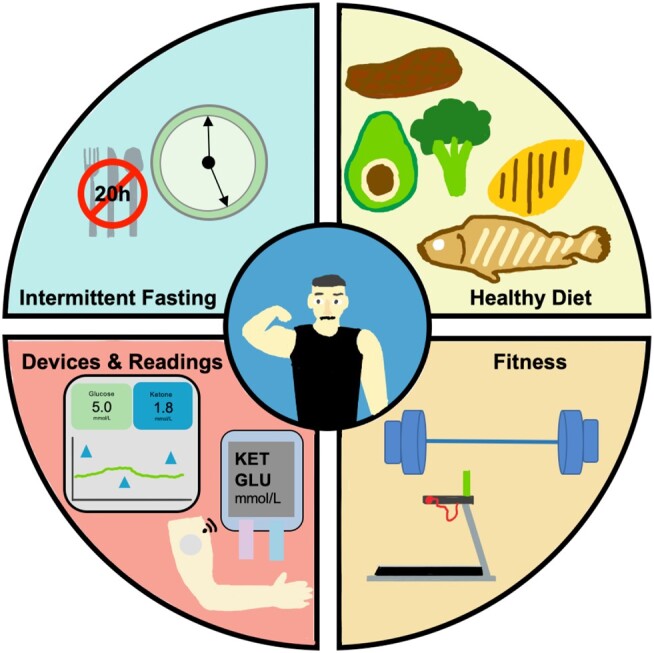
Overview of N-of-1 health optimization. The optimization workflow includes strictly adhered intermittent fasting on a minimum 20-h schedule (feeding window during dinner), biomarker readings from a suite of platforms, consistent strength and cardiovascular training, and ketogenic dietary regimen.

As the prevalence of super-aged societies continues to increase, addressing aging-related disorders such as metabolic disease and frailty will become increasingly important. This is evident given the rapid increase in national programs being initiated to prioritize preventive and population health. These include innovative platforms such as Healthier SG (Singapore), National Preventive Health Strategy (Australia), Better Health, Better Lives (Norway), and National Prevention Strategy (United States) (17–20). Given the diversity of behavioral responses to the broad spectrum of interventions available, combinatorial approaches may be needed. Classes of potential interventions currently being evaluated include conventional pharmacologic therapies, dietary supplements, diverse diet strategies, fitness regimens, as well as intermittent fasting (IF), among others (21–24). Of note, IF has been increasingly explored as an intervention to address obesity, health and performance optimization, and healthy longevity due to its accessibility and simplicity relative to other approaches as well as its core capability in helping subjects achieve ketosis—the process whereby a subject's primary energy source switches from glucose to fat breakdown (21, 25, 26). There is a vast range of biomarkers and endpoints that can be monitored against these interventions. They include standard endpoints such as blood pressure (BP), weight, and a multitude of serum panels to assess heart, kidney, and liver health for example. Other biomarkers that can be assessed include blood glucose (BG), which can be monitored using wearable devices (e.g. continuous glucose monitoring [CGM]) or handheld devices (e.g. blood/finger stick), and ketones, which can be measured using handheld devices (blood/finger stick), breath, or urine (Fig. 1). Importantly, the dynamics of these markers, as well as other indices such as the glucose-ketone index can potentially also shed further insights on health profiles as well as user behavior and personality to drive gamification strategies for behavior change (27–33).

**Fig. 2.**
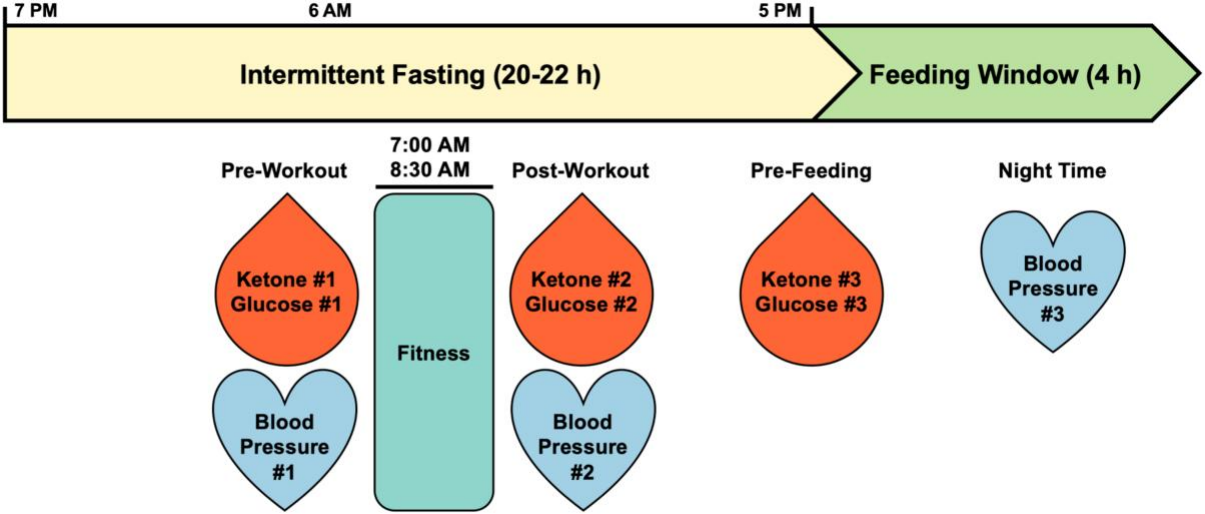
Intermittent fasting timeline. Subject N001 followed a 20/4 intermittent fast (minimum 20–22 h fasted, 4-h eating window) with morning fasted fitness regimen daily. Biomarker readings pre- and post-workout as well as prefeeding were recorded.

To assess how these factors can impact biomarker behavior and potential surrogate indicators of health status from an individualized perspective, this work reports a N-of-1 study that harnesses a suite of digital platforms to monitor biomarker dynamics in response to a regimen combining a 20/4 intermittent fast (20-h fasted, 4-h eating window), morning fasted fitness regimen daily (strength and cardiovascular), and consistent high protein (>100 g/day)/low carbohydrate (<30 g/day) diet. Biomarkers consisting of glucose and ketone were recorded prior to the fitness regimen, after the fitness regimen, and prior to opening the feeding window (Fig. 2). In addition to the longitudinal monitoring of biomarker dynamics, this work also considered the unforeseen emergence of data-enabled gamification to drive user adherence to sustain biomarker profiles. User engagement insights pertaining to device usage, biomarker monitoring, and regimen sustainability are also provided. In sum, based on the suite of available biomarker monitoring devices, capacity for longitudinal monitoring of biomarker dynamics, and resulting adherence gamification, metabolic switching served as a study endpoint. Metabolic switch refers to the process of the body's shift from glucose to ketones as a core energy source (34, 35). Metabolic switching is also being explored as a method for physiological and performance optimization, addressing obesity and other risk factors for chronic illness, as well as cardio- and neuroprotective outcomes (34, 36).

In addition to understanding N-of-1 dynamics during the metabolic switching process, preliminary findings from this work can potentially be expanded towards the development of large-scale, prospective, and interventional trials to determine if user personality can be harnessed based on individual data and biomarker profiles to drive behavioral change, with subsequent assessment of behavioral and healthcare economics outcomes at a population health level. These findings may also be applicable towards broader indications including healthy aging and longevity, sports science, and enhanced data collection protocols for AI and data science that are applicable to a wide range of prevention and treatment needs.

## Results

### Exploring metabolic switching with IF and fitness interventions

**Fig. 3.**
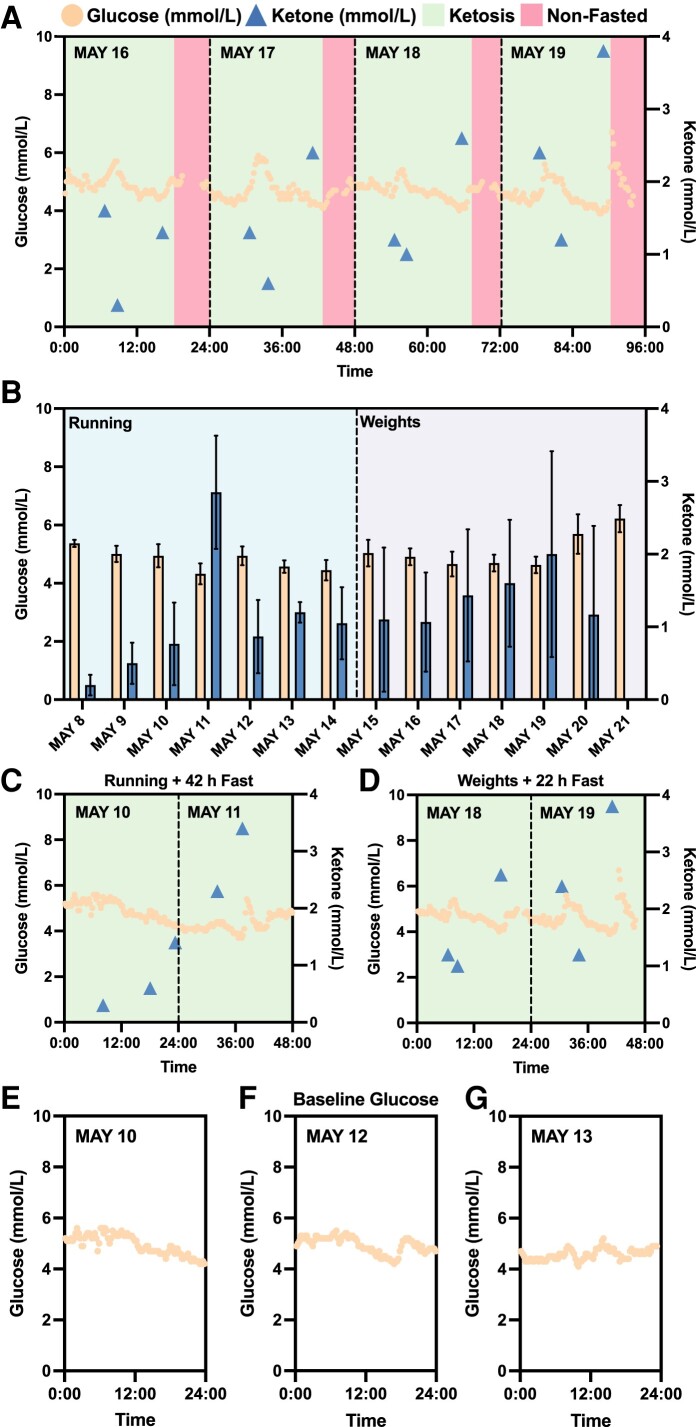
Continuous monitoring of biomarkers with intermittent fasting and fitness regimen. A) Four-day continuous monitoring of blood glucose and ketone trajectories. B) The comparison of recorded blood glucose and ketone levels with different fitness regimen (running and weights) in a 2-week period (glucose: *N* = 34–96; ketone: *N* = 2–4; daily). Wilcoxon rank-sum test determined statistically significant difference between glucose levels as a result of running and weights training (running: *N* = 600; weights: *N* = 564; *P* < 0.001). However, no statistically significance was detected for ketone levels as a result of the interventions (running: *N* = 16; weights: *N* = 18). C, D) Blood glucose and ketone profiles with interventions consisting of fitness regimen (running or weights) and intermittent fasting (42 h or 22 h) in a 2-day period. E, F, G) Subject N001's baseline glucose. Glucose and ketone levels were measured using a CGM and a finger sticking device in May 2023, respectively. Data can be found in Dataset S1 and Fig. S2.

Initially, a suite of devices was harnessed to explore the dynamics of biomarkers with the interventions of fitness regimen and IF. A CGM device was employed to measure subject N001's blood glucose levels (May 2023). In parallel, the blood ketone levels were measured pre- and post-workout as well as in the afternoon/evening immediately prior to closing the daily IF window. The fitness regimen varied daily with either cardiovascular or strength training. In Fig. 3A, the glucose and ketone trajectories were plotted against a 96-h window. Notably, the ketone trajectories demonstrated high–low–high profiles, specifically low ketone levels post-workout. However, in the afternoon or evening, blood ketone levels rebounded back to higher levels. Further examining the profiles, when ketone reached lower levels, transiently elevated blood glucose levels were observed during workouts (Fig. 3A). This may be the result of glycogen depletion during workouts.

To assess the interventions of the fitness regimen, the measured blood glucose and ketone levels were comprehensively compared following both cardiovascular (e.g. running) and strength (e.g. weights) training (Fig. 3B). The average continuously measured blood glucose levels for cardiovascular training interventions (running) was 4.75 ± 0.44 mmol/L (*N* = 600), while strength training resulted in blood glucose levels of 5.05 ± 0.70 mmol/L (*N* = 564). Wilcoxon rank-sum test determined that there is statistically significant difference between the glucose levels resulting from both fitness interventions (*P* < 0.001), while no statistically significant difference was detected for blood ketone levels as a result of fitness regimens (running: *N* = 16; weights: *N* = 18). Heavy weightlifting workouts during the second week of CGM usage may have stimulated the release of stress hormones, such as adrenaline, which may lead to increased release of glucose from the liver (37). Furthermore, fitness interventions accompanied with a 22+ h fast were also explored. In Fig. 3C and D, trajectories of either running with a 42-h fast or weights with a 22-h fast both similarly illustrated the high–low–high ketone trajectories and notably, pointed to elevated ketone levels towards the end of fasting. The elevated ketone levels may be attributed to metabolic switching from glucose depletion during 42- and 22-h fasts. These preliminary findings further confirmed metabolic switching in subject N001. The estimated HbA1c during the noted CGM timeframe was 4.8% (29 mmol/mol), indicating normal average blood glucose levels (Fig. S1). Subject N001's glucose and ketone levels are summarized in Dataset S1 and Fig. S2.

**Fig. 4.**
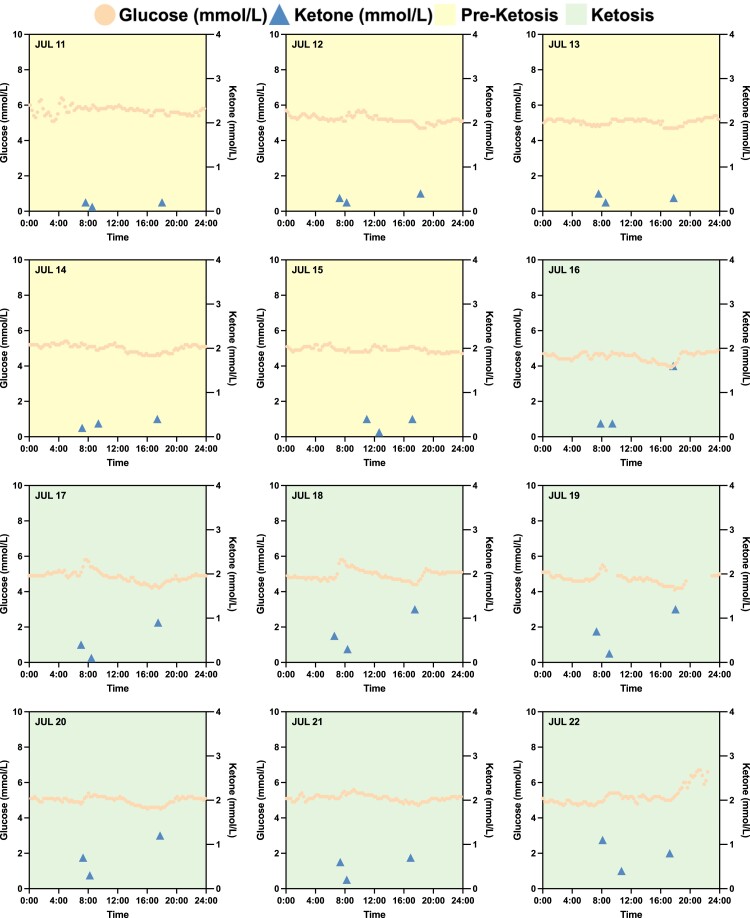
Subject N001's baseline biomarkers with intermittent fasting and fitness regimen. In a 12-day period (2023 July 11–22), subject N001 underwent IF and consistent fitness regimen in the morning. Ketosis was only achieved on July 16, after 5 days of consistent IF, and fitness and dietary regimens. Glucose and ketone levels were measured using a CGM and a finger sticking device in July 2023, respectively. Data can be found in Dataset S1 and Fig. S3.

To further investigate metabolic switching that spanned the preketosis through ketosis processes, subject N001 underwent IF for 12 consecutive days (2023 July 11–22) with consistent fitness and dietary interventions (Fig. 4). For the first 5 days, the subject's measured ketone levels were consistently below 0.5 mmol/L, representing the baseline data or preketosis stage (Fig. 4). However, on the 6th day (July 16), subject N001 achieved ketosis with the high–low–high ketone trajectories (Fig. 4). Additionally, the subject's baseline glucose levels measured using a CGM are presented in Figs. 3E–G and 4. The baseline preketosis biomarkers serve as the basis of comparison with data describing metabolic switching during ketosis. Subject N001's glucose and ketone levels are summarized in Dataset S1 and Fig. S3.

### Longitudinal monitoring of biomarkers and fitness interventions

**Fig. 5.**
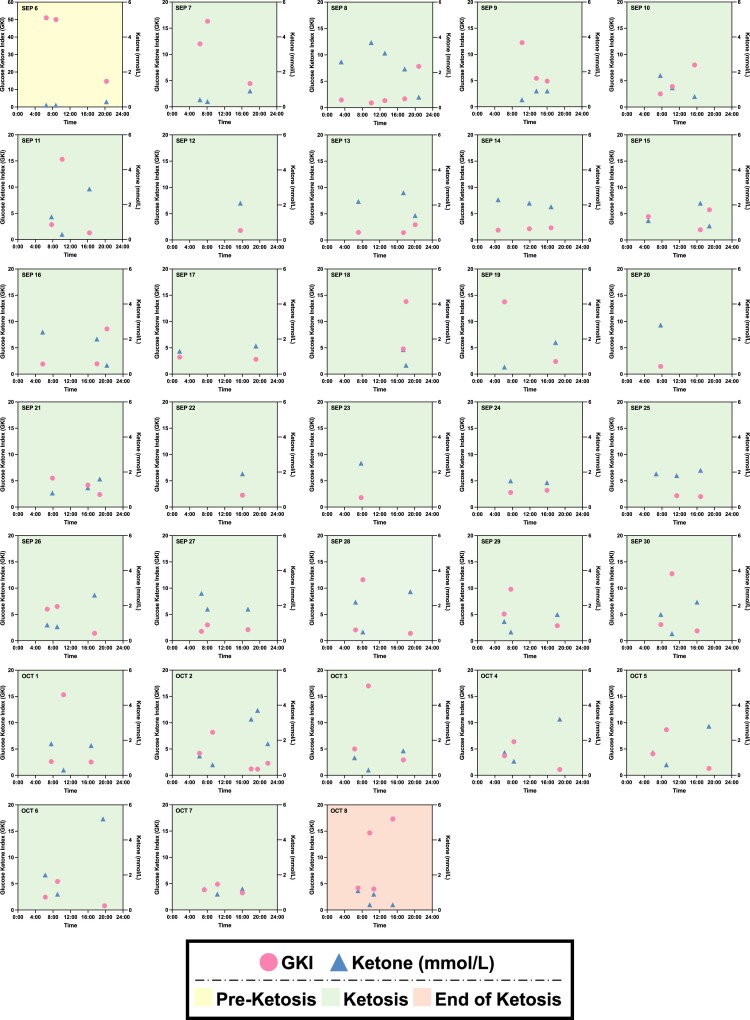
Longitudinal monitoring of ketone and glucose ketone index (GKI) trajectories for 33 days. The subject N0001 was in preketosis stage on September 6 (2023) and began ketosis the following day for 31 days. During ketosis, N001's ketone and GKI profiles were high–low–high and low–high–low, respectively. Note that some days may have been affected by regional and international travels. Glucose and ketone levels were both measured using a finger sticking device from September to October 2023. Data can be found in Fig. S4.

Subsequently, the monitoring of biomarkers was further expanded to long-term observation. Instead of CGM, N001's blood glucose levels were measured using a finger sticking device pre- and post-workout as well as in the afternoon/evening immediately prior to closing the daily IF window. The blood ketone levels, which were measured using the same finger sticking device, followed the same schedule. Normalizing the blood glucose levels to the blood ketone levels gives rise to the glucose ketone index (GKI), which can reveal the state of ketosis and overall metabolic health of an individual (27). In Fig. 5, the GKI and ketone trajectories were plotted against time for 33 consecutive days (2023 September 6 to October 8). Subject N001 was in a preketosis state on September 6 and subsequently achieved ketosis for 31 days. On October 6, the subject reached the end of ketosis state. Similar to previous observations, the ketone profiles demonstrated high–low–high trajectories (Fig. 5). In contrast, the GKI profiles displayed low–high–low trajectories, where a GKI of 3 or less indicates high level of ketosis. Subject N001's ketone/glucose levels are summarized in Fig. S4.

## No negative effects observed in 72 h IF

**Fig. 6.**
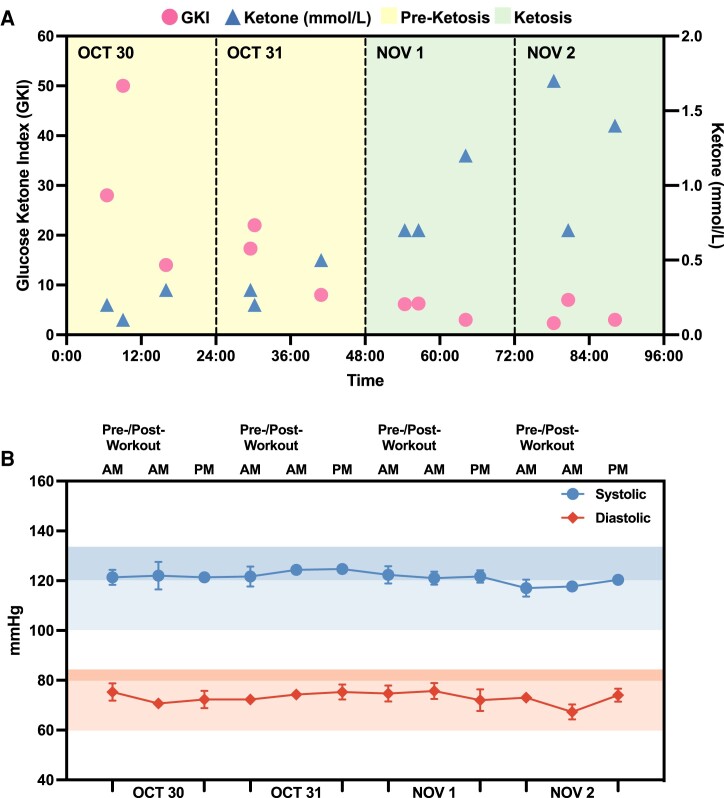
Biomarker monitoring of N001 during a 72-h fast. A) Ketosis was achieved on November 1 and high blood ketone levels were observed towards the end of the 72-h fast. B) Daily blood pressure measurements (*N* = 3) indicated no significant changes to cardiovascular health as a result of a prolonged fast. Glucose and ketone levels were both measured using a finger sticking device from October to November 2023. Data can be found in Fig. S5 and Table S1.

To monitor GKI and ketone dynamics during a long fast, subject N001 underwent a 72-h fast (October–November 2023). Blood glucose and ketone levels and blood pressures were consistently measured according to the schedule in Fig. 2. Over the 4-day period, N001's GKI gradually decreased during the 72-h fast. In the first 2 days, the GKI readings were >10, indicating that N001 was not in ketosis (Fig. 6A). However, on day 3, the subject's ketone levels substantially increased and the GKI level was observed to be below 3, suggesting a state of high ketosis. Aside from biomarker trajectories, the subject's blood pressures were closely monitored thrice daily. Subject N001's blood pressures were consistent throughout the 72-h fast (Fig. 6B). These subject-specific data suggested that a 72-h fast had no apparent adverse or negative effects on subject N001. Subject N001's ketone/glucose levels and blood pressures are summarized in Fig. S5 and Table S1.

### BP and body weight monitoring

**Fig. 7.**
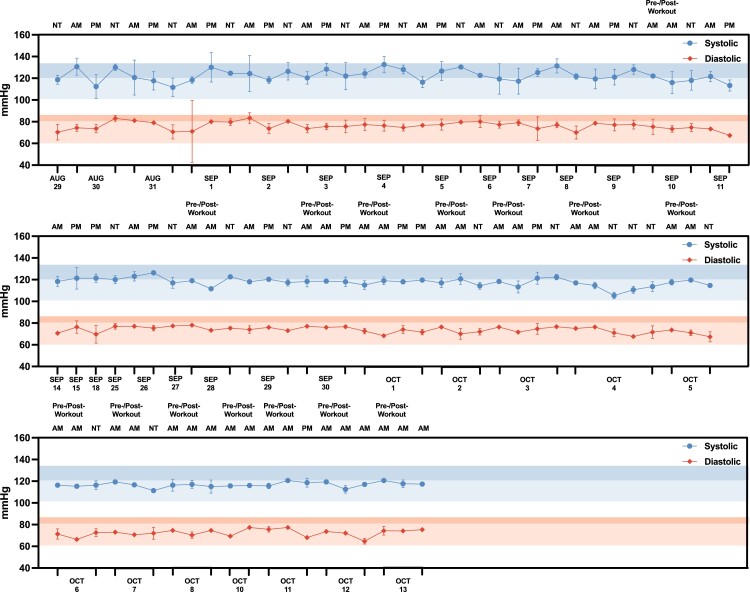
Blood pressure monitoring for subject N001. Measurements were taken pre- and post-workout in the morning (AM) and in the afternoon (PM) and night time (NT) (*N* = 3–6) from August to October 2023. The desired blood pressure benchmarks 135/85 and 120/80 are indicated in the highlighted zones, in which the darker segment represents 135/85 and the lighter segment represents 120/80 within each highlighted zone. Data can be found in Table S2.

Aside from biomarker monitoring, subject N001's BP trajectory over time (August–October 2023) was also closely monitored and recorded at least three times daily, with pre- and post-workout as well as evening measurements. (Fig. 7 and Table S2). Overall, the subject's blood pressures were mostly within the two selected benchmarks which both serve as upper limits that define elevated BP by multiple health authorities. These included systolic/diastolic limits of 135/85 and 120/80 (38, 39). Importantly, they were consistent in the duration of a month and a half. In addition, the differences between N001's BP measurements and the two respective benchmarks were determined (ΔmmHg = benchmark—measured blood pressures) to analyze potential improvement in cardiovascular health (Fig. S6). The general trend demonstrated that both systolic and diastolic measurements lowered (increased ΔmmHg) towards the end of IF along with fitness and dietary regimens. With the stricter 120/80 benchmark, N001's systolic measurements were well within range in the second half of IF while measurements in the first half were mostly out of 120 mmHg. It is important to note that there is an overall improved trend; however, this observation may be attributed by other factors including IF, fitness plans, and/or dietary regimens.

**Fig. 8.**
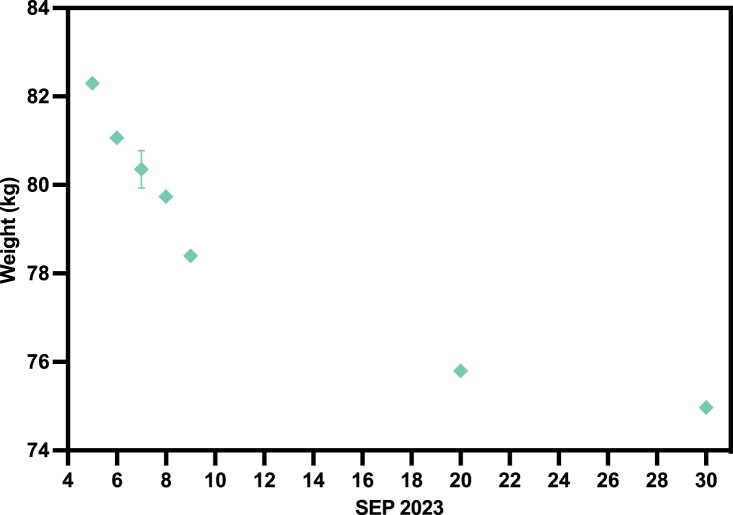
Subject N001 body weight in September 2023. The sudden drop in body weight at beginning of September was a result of a 72-h fast (September 5–8) done by subject N001 (*N* = 3–6). Data can be found in Table S3.

In the beginning of September, subject N001 underwent a 72-h fast from 2023 September 5–8. During this time and for the rest of the month, the subject's weights were recorded intermittently (Fig. 8). Fitness and dietary regimens were also regularly scheduled. As shown in Fig. 8, a 72-h fast resulted in a ∼4 kg decrease in weight. Even after the 72-h fast, the subject consistently continued with the IF, fitness, and dietary regimens, in which the subject's weight continued to decrease over time. In the beginning of September, the subject's weight was slightly above 82 kg and towards the end of September, the subject's weight was approximately 75 kg. Within 1 month, IF and the aforementioned interventions resulted in a ∼7 kg decrease in weight for subject N001. At the time of reporting, due to overall regimen adherence, the subject's weight has stabilized at 75 kg (Table S3).

## Discussion

### Initial biomarker monitoring

This N-of-1 study made use of a collection of publicly accessible devices to monitor biomarker dynamics as a function of a consistent regimen of IF, fitness, and high protein/low carbohydrate nutrition. The maintenance of these dynamics, particularly with regards to ketone trajectories, unexpectedly served as a data-driven incentive, resulting in the gamification of regimen adherence. Initial CGM and correlated ketone measurements were taken to understand the dynamics associated with pre- and post-workout glucose and ketone levels alongside the ketone measurements prior to feeding. These initial readings confirmed metabolic switching, and illuminated the early potential of harnessing data and ketone dynamics towards adherence gamification (Figs. 3–5). They also provided user-specific comparisons of the effect of cardiovascular training versus strength training on glycogen depletion towards the achievement of metabolic switching (Fig. 3). Of note, these biomarker profiles served as clear indicators that the human response to the various interventions is highly dynamic (40–44).

These observations emphasize the importance of serial health monitoring in order to sufficiently characterize a subject's health status prior to the recommendation of interventions. These biomarker dynamics also indicate the importance of understanding how an individual's biomarker levels change in response to specific interventions (e.g. fitness regimens, fasting, etc.) at fixed as well as modulated doses and intensity. These datasets would provide important information pertaining to inter- and intra-individual variation at the interface of health interventions and treatment response. In turn, this information may be helpful towards harnessing digital platforms for hyper-personalized gamification and health optimization.

### BP and weight monitoring

An assessment of BP trajectory over time revealed a modest decrease in systolic and diastolic readings. While it cannot be determined if this specific outcome is attributable to the IF, fitness, and/or dietary components of the regimen, it is evident that this regimen did not have a negative effect on the blood pressure trajectory. Previous studies have shown that IF can potentially drive beneficial BP outcomes (45–47). Furthermore, other preclinical and clinical studies have shown that adherence to set fitness and dietary regimens can have beneficial influences on both BP and weight (ref). Thus, the improvements observed in this N-of-1 study are likely a result of composite influences within the adhered regimen. An assessment of the BP during a 72-h fast (2023 September 5–8) also does not reveal a negative effect on BP trajectory, aligning with prior studies correlating acute fasts with BP outcomes (48). With regards to weight monitoring, a 72-h fast revealed a weight decrease of ∼4 kg, aligning with previously reported values (Fig. 8) (49). The weight loss trajectory tapered off over time as a steady IF regimen was implemented. It is unclear if reduced weight loss trajectory is a result of metabolic adaptation to IF regimen or reaching the lower ranges of the individual's healthy weight. Additional longitudinal studies comparing altered IF, diet and fitness regimens to further promote metabolic switching as well as comparing this N-of-1 study to other individuals with different baseline BP and weight measurements would help address this study limitation.

### Biomarker profiles and gamification

A notable outcome of this study pertained to the role of biomarker dynamics, specifically ketone trajectories, towards driving adherence to the IF, fitness, and dietary interventions. Specifically, during the initial monitoring period using the CGM and Optium Neo, ketone levels were observed to be elevated in the morning prior to fitness training, lower following fitness training, and elevated prior to feeding (high–low–high) (Fig. 5). This corresponded with observed spikes in glucose levels and depletion in glycogen stores as a result of fitness training. Mechanisms of ketone level reduction as a result of fitness training with corresponding glycogen usage have also been noted (50, 51). A further assessment of the glucose-ketone index (GKI) dynamics in this study also revealed a low–high–low trajectory. Of note, these findings with subject N001 reflect the potential of gamifying adherence to a fasting, fitness, and food regimen to maintain biomarker profiles that are indicative of metabolic switching. A number of important studies have explored the role of gamification and nudge theory to drive increased physical activity and improved outcomes for indications such as cardiovascular health and type 2 diabetes, among others. These studies have shown that user behavior insights and data from wearables can be harnessed to develop support/collaboration- or competition-based approaches to encourage sustained user engagement and adherence (29–31, 52).

More specifically, for N001, the maintenance of a dynamic biomarker trajectory unexpectedly served as a promoter of adherence to the fasting, fitness, and dietary regimen. Specifically, maintaining the high–low–high ketone profile and low–high–low GKI profile was driven by the avoidance of a high–low–low ketone profile or low–high–high GKI profile (Fig. 5). An example of an action that would result in the latter profiles include breaking the fasting with food items that are noncompliant with the clean ketogenic regimen that was undertaken (e.g. consumption of carbohydrates that exceed ketogenic diet limits). Implementation of this data- and biomarker-driven adherence approach was specifically supported by the continuous visualization and user awareness of the daily profile. These observations may support the potential exploration of achieving biomarker trajectories that reflect sustained metabolic switching as a nudging strategy. In parallel, recent studies are exploring the role of multiple classes of incentives to drive user adherence to a broad range of interventions that span medication through fitness (53). These incentives include financial through digital means (54). In the case of this reported study, both the data (which can itself serve as a potential incentive), and the trajectory of data points (which can serve as a potential nudge) can collectively provide as a potential path forward for driving user adherence. Taken together, data-driven nudging paired with sustainable incentives may also impact health optimization at-scale. A properly powered trial that properly stratifies data-responsive users may be effective in evaluating this approach towards community and population health.

### Potential health benefits

Gamification of adherence to the aforementioned fasting, fitness, and food regimen using a user-specific biomarker profile and resulting metabolic switching can potentially link a broad spectrum of factors to achieve desired health outcomes. These include but are not limited to user data, user personality, nudging, incentives, behavioral economics, and other factors towards achieving sustained behavior change. As the process of metabolic switching may have additional impact on healthy aging and physiological fitness parameters, harnessing scalable and sustainable data-guided strategies to achieve metabolic switch as part of a healthy aging regimen may warrant further study.

Following feeding, glucose serves as a core energy source, and fats are subsequently deposited in the form of triglycerides. Fasting can break down triglycerides into fatty acids that are subsequently metabolized to ketone bodies in the liver via ketogenesis (21). Metabolic switching refers to the process of converting from glycogen depletion and fat storage to fat mobilization and liver-based conversion of fatty acids into ketone-driven energy sources (34). Preserving muscle mass is one of the potential outcomes of metabolic switching. In addition, studies have sought to examine whether interventions such as IF drive health benefits by addressing obesity, or due to metabolic switching (55). Findings have suggested that harnessing ketones as an energy source may have protective functions against oxidative stress and can potentially be cardioprotective (56). Additional studies have also explored the neuroprotective function of ketones as well (57).

A clean Mediterranean-based ketogenic dietary regimen was strictly followed by subject N001. For example, chicken and olive oil served as the primary source of proteins and fats, respectively. In contrast, a standard American diet (SAD) is conventionally energy-dense, but the composition of the regimen can also be high in saturated fats (58). The associated risks of SAD may include cardiovascular diseases, diabetes, and obesities. A recent study investigated the effects of the ketogenic diet (<30 g carbohydrate/day) and SAD in 30 patients diagnosed with metabolic syndrome (MetS) (59). Participants were randomly assigned to one of three research groups: ketogenetic diet with no exercise, SAD with no exercise, and SAD with exercise. In a 10-week period, patients who followed the ketogenic diet resulted in significant reductions in weight, body fat, BMI, and HbA1c (59). Additionally, the ketone levels in the ketogenic group also resulted in significant increases over the course of the study (59). In an 8-week randomized controlled trial (NCT00166088), obese or overweight US adults were randomly assigned to three arms: (i) Mediterranean diet, (ii) high-fat American diet supplemented with fish oils, walnuts, and grape juice, and (iii) high-fat American diet (control arm) (60). In contrast to the control (high-fat American diet), subjects in the Mediterranean diet arm had significantly greater weight loss and lower cholesterol (60). Additional trials including the PREvencion con DIeta MEDiterranea (PREDIMED) trial have reported beneficial effects of Mediterranean diet in reducing the risks of cardiovascular diseases, diabetes, and obesity (61–63). Furthermore, achieving ketosis relies heavily on limiting carbohydrate intake. It is well established that exceeding thresholds for carbohydrate intake will preclude ketosis (59, 64, 65). As such, measuring metabolic switching via blood ketones can serve as a clear and unambiguous marker that is unlikely to achieve passively (21, 34, 55). This further accentuates the importance of pairing fasting with the right dietary regimen (e.g. Mediterranean-based ketogenic diet) to support and sustain metabolic switching.

Importantly, this study showed that due to dynamic changes in glucose and ketone levels at different time points, assessing the respective biomarker levels or GKI at single time points may not provide a comprehensive view of a person's health status.

As continued studies illuminate the potential benefits of metabolic switching, developing strategies that help individuals achieve the metabolic switch may be instrumental in advancing preventive healthcare, addressing age-related diseases and improving general health outcomes.

### Implications for artificial intelligence and data science

The potential for continued, large-scale trials pertaining to metabolic switching-based endpoints can potentially be harnessed to derive population-based and individualized combination regimens of fasting, fitness, and food regimens. However, realizing these outcomes requires the collection of suitable datasets from such trials. Data are core driver of the development and validation of AI models. In standard care, data are often based on fixed dose at specific time points, or snapshots of patient response. When longitudinal data is collected, serial patient response is noted, but still at fixed dose. Previous work has illuminated a potential need to understand each patient's response longitudinally as it correlates with variable treatment magnitude (dose, intensity, etc.). Conceptually, when designing regimens with AI, it would be essential to collect user data that modulates the intensity of the various inputs of the regimen (e.g. fitness intensity, supplement dosage, etc.) while also assessing dynamic nature of biomarker profiles in response to this modulation in order to accurately and comprehensively capture individual performance. This has led to early trials that dynamically modulate drug dosing over time—a stark different from traditional, fixed-dose administration (66–70). Aside from clinical implications of impacting treatment outcomes, these studies may also pave the way for new datasets that provide enhanced insights into how best to dynamically adjust treatments for possible hyper-personalization. In the context of this reported study, while it is widely acknowledged that patients are different from one another, each patient also evolves over time. Therefore, further exploring the concept of evolving treatment alongside each patient dynamically may also be warranted. From a population-scale and healthy individual perspective, understanding how combination regimens comprised of inputs such as IF, fitness, and food regimens impact biomarker dynamics coupled with stratifying for user populations that can be motivated by their own data (such as these dynamics) to drive adherence can potentially lead to behavioral change outcomes that improve population health at-scale.

### Health economics considerations

The findings from this work also draw attention to health economics, nudge, and health behavior considerations. Using hypertension as an example, less than 25% of patients in the United States maintain controlled BP (71). These statistics primarily result from noncompliance due to a perceived lack of benefits (72), and unsuitable interventions with associated side effects. To overcome these challenges, studies have demonstrated that adequately addressing patient engagement can potentially improve compliance, and consequently improve clinical outcomes with substantial cost savings (73, 74). Furthermore, the annual cost of drug-related morbidity and mortality resulting from suboptimal pharmacologic intervention was estimated to be nearly $530 billion (75), constituting 16% of the total US healthcare expenditures in 2016 (75). Addressing the economic impact associated with patient noncompliance and suboptimal therapies in the healthcare system is therefore of considerable importance. These challenges draw attention to the need to develop user-centered interventions that align with an individual's preferences and personality, motivation styles, and decision-making patterns, and subsequently incorporate various components such as behavioral nudges, gamification, feedback systems, predictive analytics, and diverse incentive structures (such as tangible rewards, social recognition, or intrinsic satisfaction) (Table 1). These mechanisms, individually or collectively, can lead to better patient engagement and improved sustainability of regimens (76–82).

**Table 1.** N-of-1 health user engagement.

Platform	Function	User considerations	Summary
Wearable (Libre)	Not primarily used to track fasting blood sugar due to low carbohydrate diet Occasionally used to track blood glucose impact of certain foods Tracked glucose trajectories before, during, and after fitness training to understand glycogen depletion Blood glucose monitoring during longer term intermittent fasting (e.g. 72-h)	Possible to use 2-week period to prioritize users who may respond to receiving their days to adhere to dietary and fitness regimens Helpful for comparing differences in glucose profiles/glycogen depletion between strength training vs. cardio training Tapping the phone to sensor and haptics were helpful for rapid and immediate tracking of glycogen depletion and adherence	Device helpful for tracking serial glucose dynamics and potentially using GKI to gamify user adherence to health interventions In addition to serial glucose monitoring, device may be helpful for subjects to monitor immediate glycogen response to fitness regimens
Finger Stick (Optium Neo)	Primarily for serial ketone measurements before and after fitness training and prior to breaking fast Economical alternative to glucose monitoring in absence of continuous glucose monitoring (CGM) For measurement of glucose-ketone index (GKI)	Sticking procedure did not impede adherence to taking measurements for both ketone and glucose measurement Fidelity to obtaining data points rendered any discomfort virtually unnoticeable Obtaining readings became regular part of daily health monitoring routine	Serial finger sticking enables health insights that may motivate a subset of users to sustain healthy behavior Data obtained from serial biomarker readings may result in unique data sets that can help with hyper-personalized health optimization
Regimen (fitness and dietary)	Consistent cardiovascular and strength fitness regimens to understand glycogen depletion Intermittent fasting regimen included minimum 20-h fasted and 4-h feeding window (20/4) and rare instances of 18/6 to accommodate flights and family considerations (on average ≤ 1/week) Clean keto diet implemented, with lean protein, green leafy vegetables, nuts, and only olive oil for cooking Fasting-driven ketosis served as an endpoint for the dietary regimen	Observing switch from high ketones (prefitness) to low ketones (postfitness) was driver for fitness adherence Maintaining ketone rebound prior to feeding window was driver for dietary regimen adherence Regimen adherence maintained after data collection cut-off Images of dietary intake were recorded and caloric intake was not recorded to mitigate excess time spent on maintaining workflow	Sustaining IF, fitness, and dietary intervention requires discipline, but biomarker dynamics may support adherence for motivated users Regimen and monitoring were sustainable throughout study amidst scheduling and travel considerations

Rapidly aging societies also necessitate new approaches to promote healthy behaviors at the population level. For instance, in the United States, 75% of healthcare costs are allocated to treating preventable chronic conditions due to poor dietary and lifestyle choices (e.g. insufficient physical activity) (83). The study reported here raises a pivotal question on whether personalized health interventions supported by digital health can be effectively implemented at the population health level. Additional factors that need to be addressed include cost-effectiveness and acceptable cost per additional outcome in accordance with the standards set by individual countries.

As an example, Singapore previously launched a substantial public health intervention to enhance population-level physical activity. The primary components of this intervention included the distribution of fitness trackers, the provision of redeemable rewards, and the incorporation of gamification elements. This initiative led to a rise in the mean number of participants' daily steps during the pre–post challenge periods. A 2017 national population survey also revealed that incidental physical activity among adults increased from 5% in 2010 to 14% in 2017, suggesting a potential lasting impact (84). Beyond physical activity, evidence on the cost-effectiveness of population-wide dietary interventions is emerging, with economic evidence in favor of national strategies for reducing sodium intake (85).

The scalability and cost-effectiveness of such population-wide interventions are influenced by many factors, including the nature of the intervention, the targeted population, and its various components, as well as education and delivery mechanisms (86, 87). The study reported here illuminates the need for behavior and technological interventions to be paired with economic evaluation at population scale. These evaluations should not only assess the various components often required, such as the suite of digital platforms described in this article, to harness user personality and behavior but also evaluate their effectiveness in reducing disparities and reaching the most vulnerable populations.

### Community health considerations

This N-of-1 study is a prime example of how digital health tools could be used to empower health-related self-monitoring and self-regulation in the community. In this case, digital tools were used to obtain surrogate biomarkers of metabolic switching during fasting, which provided personalized biofeedback, motivated continuous progress monitoring, and informed one on how to manage and optimize metabolic health. Given the economic benefits of preventive healthcare (88), healthcare systems have increasingly tried to integrate digital tools into healthcare service delivery models, especially in bridging the care continuum from an acute to a community setting (89). This includes the use of CGMs in the primary care setting to remotely monitor the blood sugar control of community-dwelling patients with diabetes (90), and diet monitoring apps to promote weight management among people with overweight and obesity (91). However, the adoption of such initiatives among key stakeholders like clinicians and patients remains challenging largely due to the lack of appropriate implementation strategies (90). More emphasis could be placed on translating evidence-based interventions into real-world implementation by first identifying the implementation barriers, facilitators, and gaps using a systems approach. Common implementation frameworks used include the Reach, Effectiveness, Adoption, Implementation, and Maintenance framework (92), the Precede-Proceed model (93), Practical, Robust Implementation and Sustainability Model (PRISM) (94), and the Consolidated Framework for Implementation Research (CFIR) (95).

A policy-level push such as the HealthierSG initiative could be necessary to drive a population-based digital preventive health model using a systems approach. This way, population health concerns not only the healthcare professionals but everyone in the community through various means. For example, community partners such as grassroots leaders and primary care providers could partner up to play a more defined role in promoting the ownership of one's health through healthy behaviors. Currently, grassroots leaders have regular meetings on neighborhood security, town facilities and amenities management, resident feedback, and social cohesion. To raise awareness of residence health, resident networks could play a major role in promoting screening programs and creating a culture of healthy living.

Nurses who form the largest proportion of the healthcare workforce with the most contact with patients at the bedside are in the best position to promote health behavior change postdischarge by empowering patients with the knowledge and skills to do so (96). With the increase in technological applications within the healthcare sector to augment the efficiency and productivity of healthcare service delivery, nurses could also play a role in patient advocacy, to promote facilitators and reduce barriers to behavior change. For example, nurses could be trained to identify patients with excess body weight and provide the patients with healthy weight loss advice beyond the common general advice to exercise more and eat less. More useful advice would include information such as various healthy diet patterns, how to evaluate the quality of food items, and suitable exercise regimes for patients of different health statuses (e.g. one with excess body weight may not be suitable for high-intensity full-body training initially due to excess stress on the knees). With the increasing accessibility, customizability, and user-friendliness of technologies available, community nurses with an understanding of each patient's needs and the community resources available, could co-create digital strategies with community partners to promote a healthy lifestyle. An example would be to design, develop, and implement a convenient digital solution for self-monitoring, self-regulation, and behavioral nudging. During the COVID-19 pandemic, the need for contactless information transfer accelerated the digital transformation in Singapore, especially in the healthcare and finance sectors. Moving forward, health promotion initiatives could leverage on the established digital infrastructures and connectivity for secured, consistent, and efficient information transfer and storage to improve preventive health.

### Validation and regulatory considerations

While this reported study pertains to N-of-1 findings and insights, its outcomes may inform downstream regulatory/validation considerations. For example, from a clinical trial design perspective, harnessing metabolic data may serve as a trial incentive. In addition, longitudinal data can potentially provide individuals with personalized feedback on their progress. The ability to visualize trends and the sense of data ownership may serve as a powerful motivator for participants, increasing trial adherence. The same data informs researchers about individual responses to interventions. By understanding behavioral patterns, researchers can tailor interventions to enhance adherence. On a related note, longitudinal data can further inform adaptive trial designs, allowing real-time adjustments based on observed trends and behaviors.

Concerning trial recruitment, a potential participant might be more inclined to give consent to a trial with continuous access to their own data, as it increases the transparency and granularity of the treatment effects. Upon consenting, participants understand they will be able to appreciate the rationale of the decisions taken, based on real-time data, and potentially witness how the advantages of the intervention materialize throughout the course of the research.

The ability of wearables to capture longitudinal data during the course of a typical daily routine can facilitate long-term follow-up studies, providing insights into the sustained effects of interventions. Participants might feel more comfortable keeping engaged in a trial where clinicians—and themselves—can extensively monitor their metabolism. This can also contribute to fulfill real-world evidence (RWE) regulatory requirements. RWE refers to clinical evidence about the usage and potential benefits or risks of an intervention obtained outside of a research setting. Although typically restricted to postmarket studies, there is recent interest in the use of RWE to aid regulatory decision-making in the premarket phase (97). Additionally, a higher personal involvement and perception of relevance among participants in this type of trials aligns with regulatory trends toward more patient-centric and flexible trial designs (98).

From a regulatory perspective, agencies/bodies may examine the ethical implications of using biomarker trajectory-based gamification elements to promote adherence. Building a *quantified-self*—the digital representation of the body with self-tracked data—can become addictive (99). The user generates graphics that, in addition to showing their performance, have an esthetic value (100). Instead of an incentive to improve, some subjects might find a new domain for unhealthy competition (101). In chronic disease monitoring, some trial participants might worry about underperforming data (102).

### Study limitations

The reported study was based on a single subject, and was not intended for the derivation of population-scale conclusions pertaining to biomarker, dietary, IF, fitness, weight management, or other readouts. Additional factors such as demographic-specific parameters for safe fasting, fitness, and food regimens should also be considered. As the user feedback and comments were also obtained from a N-of-1 study, this work solely provides single subject insights following the specific regimen as noted. It would also serve beneficial to build upon the current study by expanding the variety of biomarkers monitored, allowing the study to take on a more holistic approach. An example would be to further investigate cardiovascular risk through observing apolipoprotein B and A-I (apoB and apoA-I) ratios or conducting lipid panels to longitudinally monitor subject cholesterol and triglyceride levels to serially assess coronary risk considerations (103). Additional biomarkers pertaining to healthy aging that may be ideal to examine in future studies also include, but are not limited to, estimated globular filtration rate through cystatin C measurements alone or in combination with creatinine (predictive of kidney function) (104, 105), total bilirubin or the combination of gamma-glutamyl transferase and alkaline phosphatase (predictive of general liver function) as well as pro-inflammatory markers such as plasma interleukin-6 and tumor, necrosis factor α (predictive of natural ageing progress) as well as other longevity markers (106–109). Furthermore, including additional platforms (e.g. Apple Watch, Fitbit) to monitor daily activities and measuring daily nutrition patterns may address potential confounding influences of varying daily patterns. Additional digital readouts may include strength, cognitive performance, and other physical performance assessments. While every effort was made to ensure consistency of biomarker monitoring timeframes and frequency, a properly powered human trial with adequate trial support and monitoring frequency and parameters (e.g. BP, weight) may ensure that population scale datasets are fully populated. Importantly, safe fasting, fitness, and dietary intervention is essential. Therefore, it should be noted that the aforementioned IF, fitness, and dietary regimens should only be undertaken under the approval and guidance of a licensed medical professional/physician.

In conclusion, this study explored the possible role of data and biomarker trajectory to gamify adherence to a fitness, fasting, and food regimen that drives metabolic switching, a process that may be associated with cardiovascular and neuroprotection. While achieving metabolic switch via interventions such as IF may be a promising objective at the individual and population levels, the ability to achieve metabolic switch may also potentially decrease over time during the course of aging (35). This observation may serve as an impetus to develop combinatorial approaches that integrate technology, fitness, fasting, dietary guidances, serial monitoring of biomarker dynamics/data, nudging, incentives, and behavioral interventions to help subjects achieve and sustain metabolic switch. The observations of the highly dynamic ketone and glucose profiles from this study, while at an N-of-1 level, emphasized the importance of serial biomarker monitoring in order to accurately and comprehensively assess health status. These insights may subsequently inform the design of larger prospective trials to address a number of endpoints. These include identifying digital health and data-responsive subjects, lifestyle-driven improvements in cardiovascular health at-scale and the potential development of alternate data collection protocols for downstream clinical decision support and AI/ML-driven approaches to health regimen optimization design. Ultimately, addressing these aforementioned parameters in an integrative manner can potentially realize actionable roadmaps to improve a broad range of applications that span community and population health, to healthy aging and longevity research, among others.

## Materials and methods

### Institutional Review Board (IRB) approval and informed consent

Institutional Review Board (IRB)/ethics approval was obtained for the study (NUS-IRB-2023-801). The study is regulated under Singapore's Human Biomedical Research Act (HBRA) due to (i) the intention of studying the performance or endurance of human individuals; and (ii) the use of individually identifiable health information. Informed consent was obtained from the subject (Co-author and Principal Investigator D.H.).

### Subject N001's overall health

Prior to this study, the subject underwent relevant clinical testing pertaining to renal, liver, and metabolic health. The results indicated that subject N001 was healthy and suitable for this study. No inter-current illness (e.g. viral infections) was reported by the subject. The subject's observed HbA1c level was observed to be 4.8% (29 mmol/mol), indicating a favorable metabolic health profile (Fig. S1). In addition, the dynamic metabolic switching process as a function of fitness and fasting while observing a healthy dietary regimen is also a potential indication of a favorable metabolic health profile.

### Intermittent fasting

Except where noted, IF regimens consisted of a 20/4 schedule (minimum 20 h fasted, 4 h eating window). This IF schedule was designed upon what was most suitable and sustainable for subject N001. However, an 18/6 (on average ≤1/week) was utilized to accommodate flights and family considerations. Every effort was made to maintain a consistent fasting window, and adherence was generally positive as reflected by the blood glucose and ketone readouts. Where necessary and possible due to international and/or regional travel, the feeding window was adjusted to lunchtime to account for time zone changes. When on international flights, both meals were served simultaneously to maintain fidelity to eating windows.

### Dietary regimen

As ketone measurement was a core output of this study, a clean Mediterranean-based ketogenic dietary regimen, which has been known to improve cardiovascular health, was followed (110–113). Moreover, the anti-inflammatory and antioxidant effects of Mediterranean diet may lead to a favorable metabolic environment that improves the outcomes of ketogenic dietary regimens (113, 114). Protein was obtained primarily from chicken. Fats were primarily derived from olive oil, avocados, pecans, chia seeds and pumpkin seeds. Fiber was obtained from leafy green vegetables (e.g. Primarily kale, spinach, and arugula). A multivitamin containing vitamins A, C, D_3_, E, and K was taken daily (Centrum; CEM-75792). No alcohol was consumed during the study. During fasting periods, electrolytes, water, tea, and coffee were allowed. In a local context, the coffee and tea were only taken with no sugar and no milk (Kopi O Kosong and Teh O Kosong). The dietary regimen was consistently photographically recorded for documentation purposes (Fig. S7). However, caloric intake was not recorded.

### Fitness regimen

Fitness regimens consisting of either cardiovascular or strength training were designed to best suit subject N001 and more importantly, to improve N001's functional ability, cardio-metabolic health, and well-being (115, 116). Previous research has shown favorable results when integrating IF and exercise, including improvements in body weight, BP, and lipid profiles (117). Additionally, a study had also demonstrated that combining a Mediterranean diet with exercise can lead to better health-related quality of life by significantly enhancing physical and functional fitness and reducing cardiovascular risk factors compared to focusing solely on dietary changes (118). For fitness regimens during continuous glucose monitoring (CGM, Abbott Freestyle Libre), the first week consisted of daily cardiovascular training (∼1 h) via the same running route. The second week consisted of daily strength training (∼90 min, each day focused on a specific muscle group). For regimens corresponding to finger stick measurements, weekly regimens were generally comprised of 5 days of strength training and 2 days of cardiovascular training. Every effort was made to commence fitness training at 7:00 AM daily. For both cardiovascular and strength training regimens, in the event of time restrictions/scheduling considerations for N001, both regimens were shortened to a minimum of 30 min to ensure regimen alignment. Nonetheless, despite the running regimens being comparatively shorter in duration, they consistently reached Zone 4 for heart rate, and strength training regimens reached Zone 2.

### Blood glucose and ketone monitoring

Blood glucose monitoring was conducted using both continuous glucose monitoring (CGM, Freestyle Libre, Abbott Laboratories, Ltd.) and finger sticking (Optium Neo, Abbott Laboratories, Ltd.). Manufacturer specifications were followed for Libre and Optium Neo usage. Ketone measurements were also conducted using the Optium Neo. With regards to Optium Neo-based ketone and glucose monitoring, unless specified, readings were taken in the morning prior to fitness training, after fitness training, and immediately before starting the eating window. The glucose and ketone finger stick regimen were consistently photographically recorded for documentation purposes (Figs. S2–S5).

### BP monitoring

BP was monitored at multiple time points, consisting of morning, mid-day, and evening. For each timepoint, three readings were taken. It should be noted that where stated, the monitoring device was changed to enable confirmation of the accuracy in arm position. Models used were the Microlife Gentle+ arm cuff monitor and the Omron 7 series wrist cuff Intellisense monitor.

### Weight monitoring

Bodyweight measurements were taken daily during the initiation of a 72-h fast, with follow-on measurements taken at subsequent timepoints (Omron KaradaScan). These readings served as readouts for regimen compliance.

### Statistical analysis

Selected biomarker and weight measurements were taken at least three times at a given time point, and the SD were determined from the average of all three measurements either at a given time point or in a day. The distribution of measured glucose and ketone levels was tested using the Shapiro–Wilk normality test. The statistical significance of glucose/ketones from different fitness regimens was determined using Wilcoxon rank-sum test.

## Supplementary Material

pgae214_Supplementary_Data

## Data Availability

Subject N001's data can be found in the supplementary information (Figs. S1–S7 and Tables S1–S3) and supplementary data (Dataset S1).
