# Supplementary material for: N-of-1 health optimization: Digital monitoring of biomarker dynamics to gamify adherence to metabolic switching
Source: PNAS Nexus. 2024 May 30;3(6):pgae214. doi: 10.1093/pnasnexus/pgae214 (PMC11179112; doi:10.1093/pnasnexus/pgae214)
Supplement: pgae214_Supplementary_Data [file pgae214_supplementary_data.zip › PNASNEXUS-PNASNEXUS-2024-00143R-s01.docx]

**
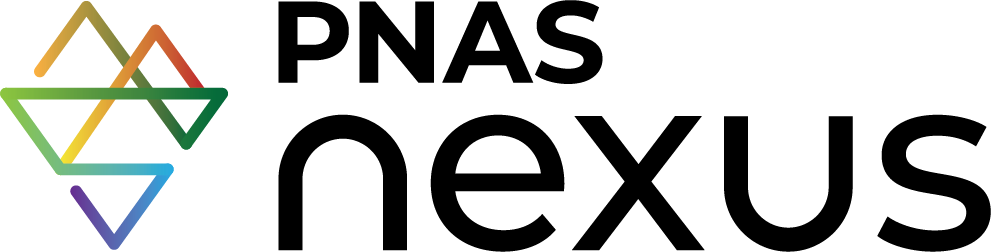
**

**Supplementary Information for**

N-of-1 Health Optimization: Digital Monitoring of Biomarker Dynamics to Gamify Adherence to Metabolic Switching

Peter Wang, Xavier Tadeo, Han Shi Jocelyn Chew, Yoann Sapanel, Yoong Hun Ong, Nicole Leung, Edward Kai-Hua Chow, Dean Ho

Corresponding Authors: Dean Ho, Peter Wang, and Xavier Tadeo

**Email:**  [biedh@nus.edu.sg](mailto:biedh@nus.edu.sg) (D.H.), [lsipww@nus.edu.sg](mailto:lsipww@nus.edu.sg) (P.W.), [lsixtc@nus.edu.sg](mailto:lsixtc@nus.edu.sg) (X.T.)

**This PDF file includes:**

Figures S1 to S7

Tables S1 to S3

**Other supplementary materials for this manuscript include the following:**

Dataset S1


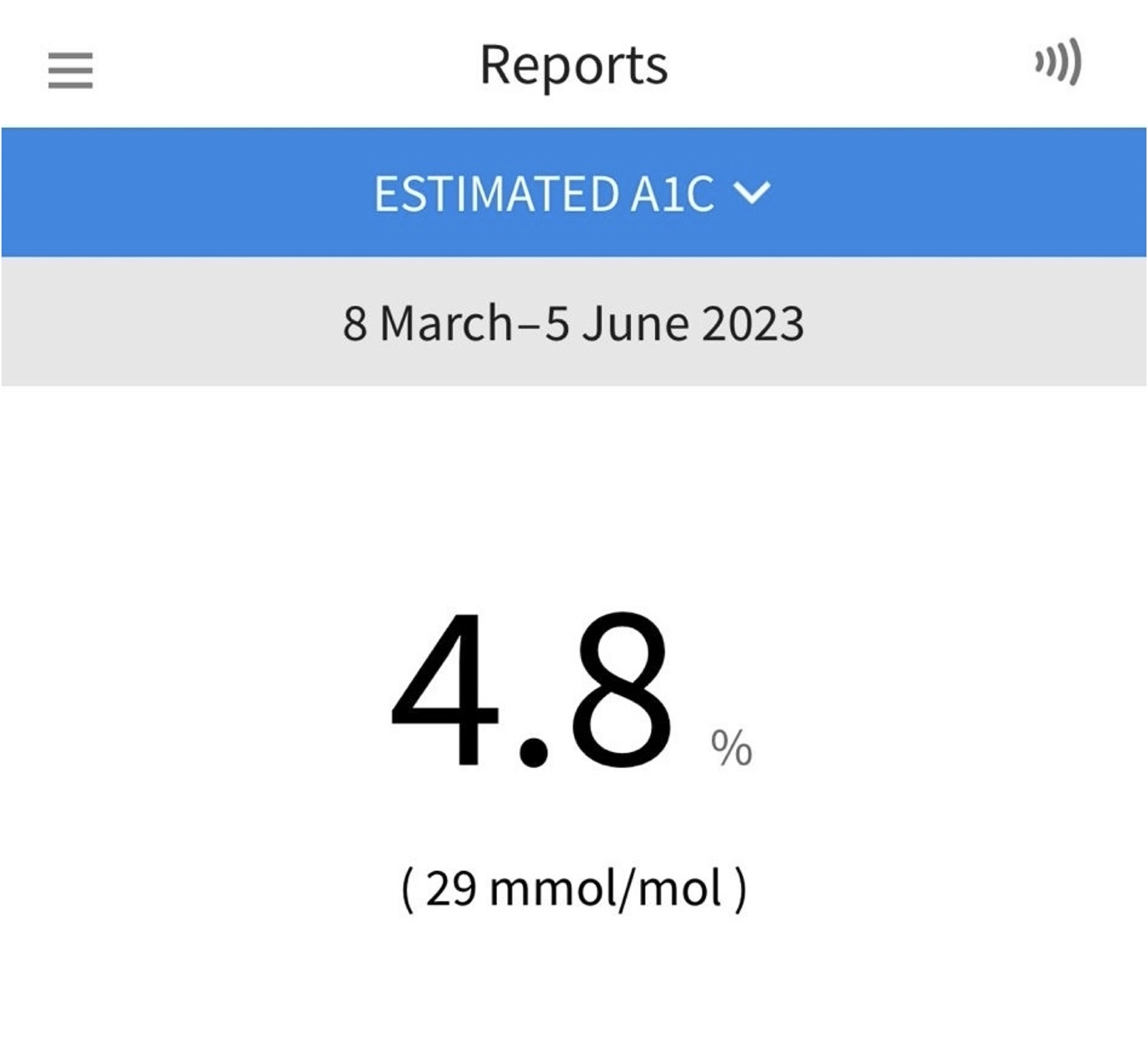


**Fig. S1.** Subject N001’s estimated A1C. The estimated A1C during the noted CGM timeframe was 4.8%, or 29 mmol/mol, indicating normal average blood glucose levels.


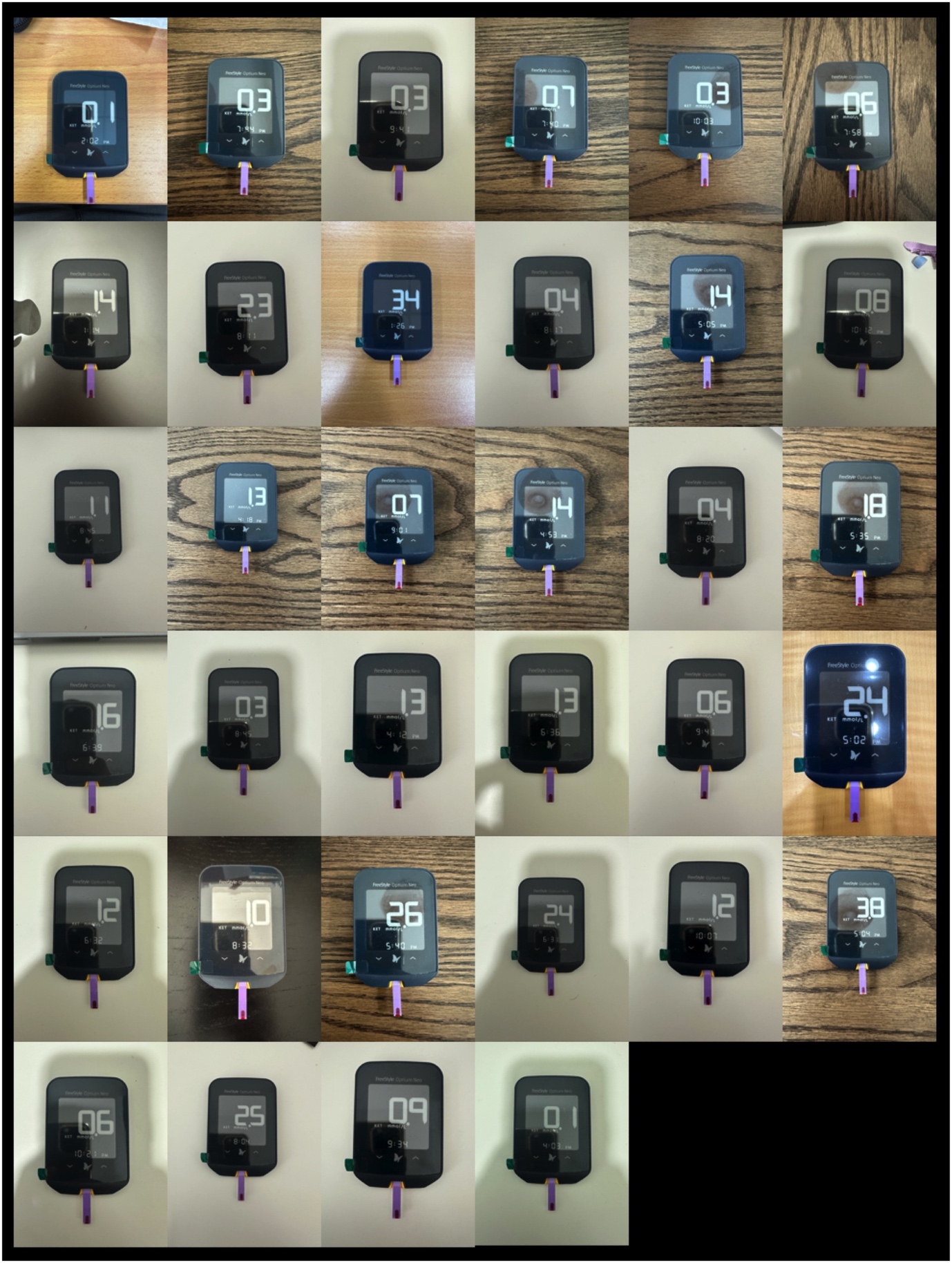


**Fig. S2.** Subject N001’s ketone levels. Photos of recorded ketone levels from MAY 8 to MAY 20, 2023.

**
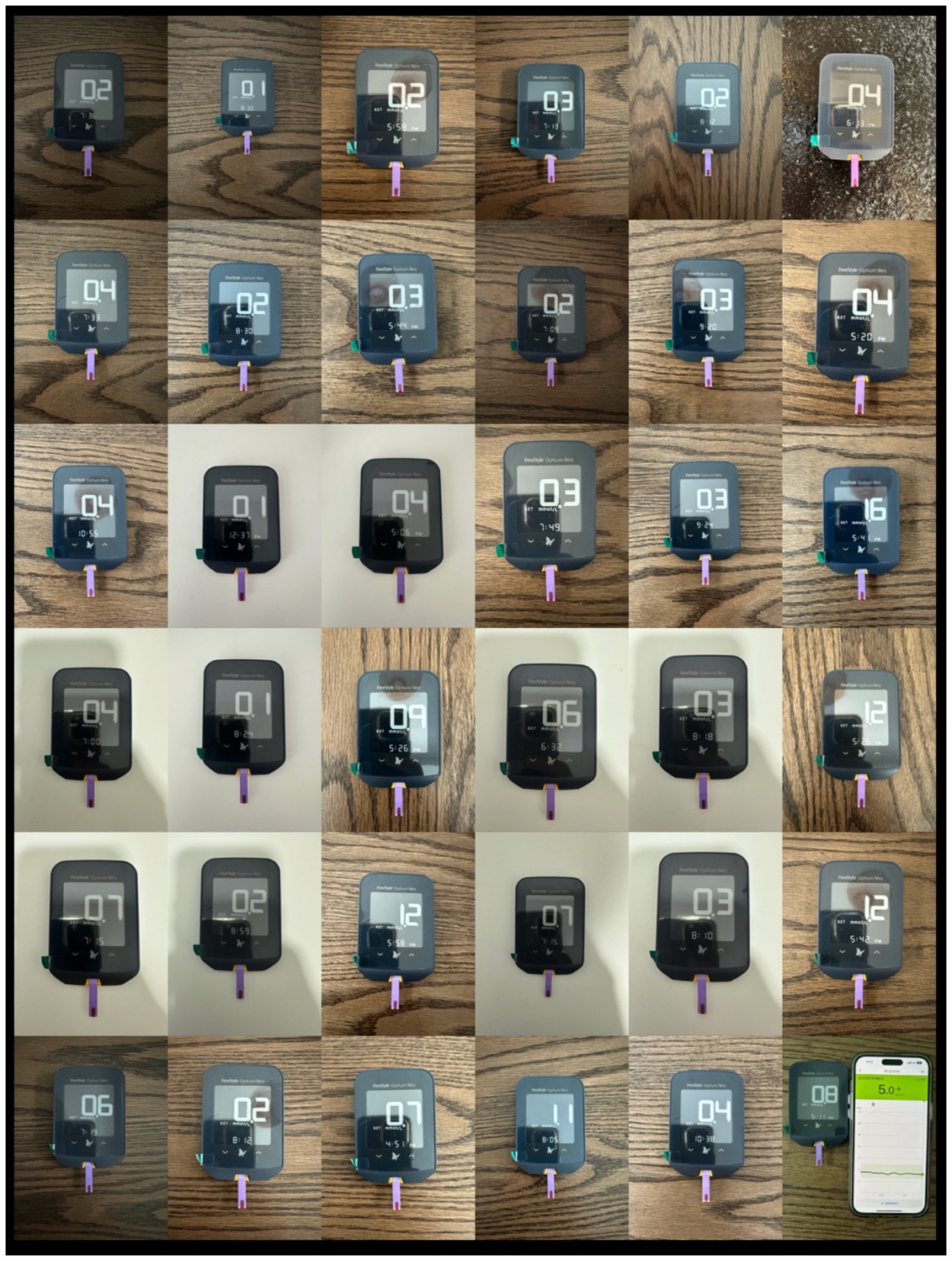
**

**Fig. S3.** Subject N001’s ketone levels. Photos of recorded ketone levels from JUL 11 to 22, 2023.

**
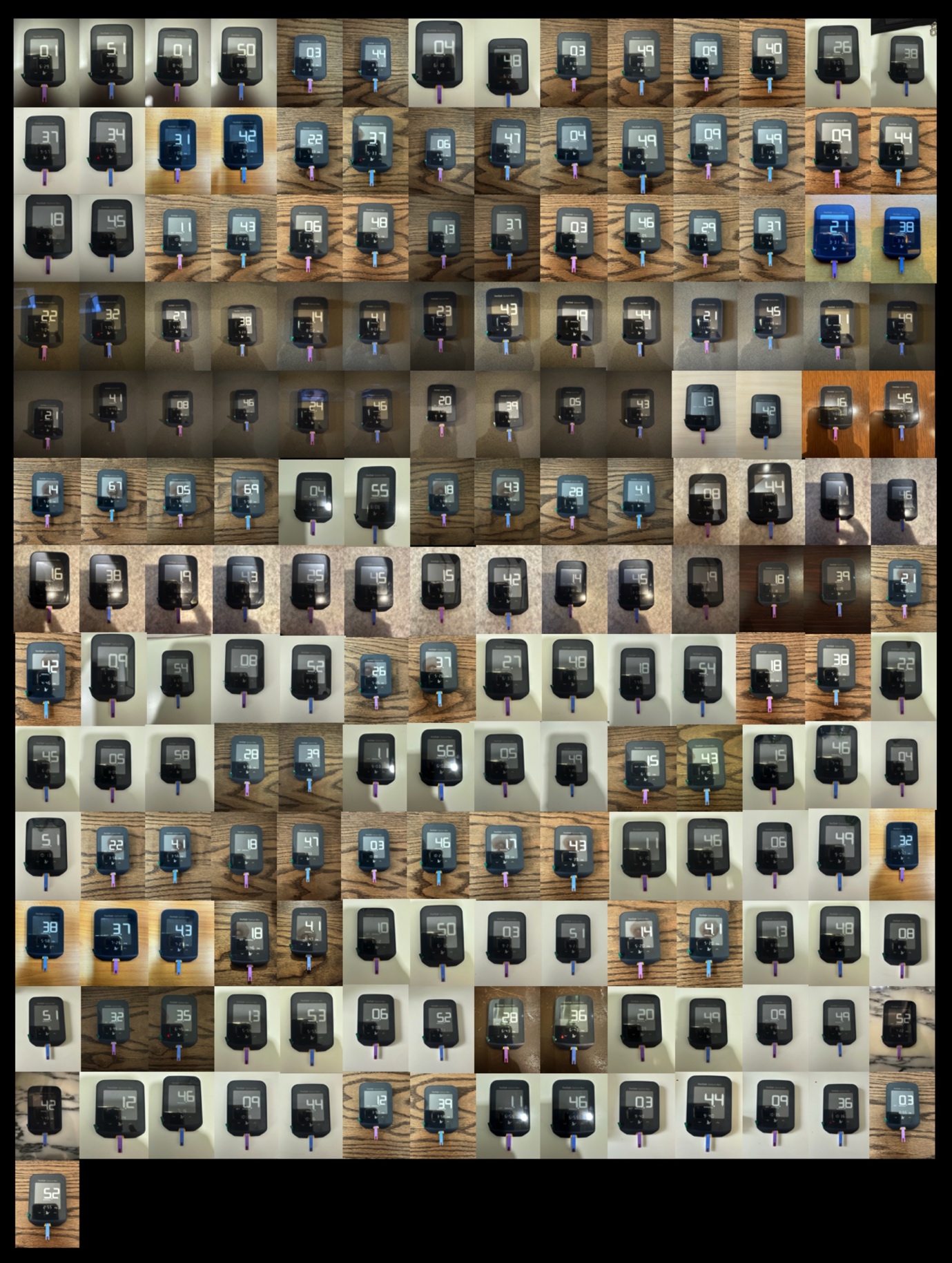
**

**Fig. S4.** Subject N001’s ketone and glucose levels. Photos of recorded ketone and glucose levels from SEP 6 to OCT 8, 2023.


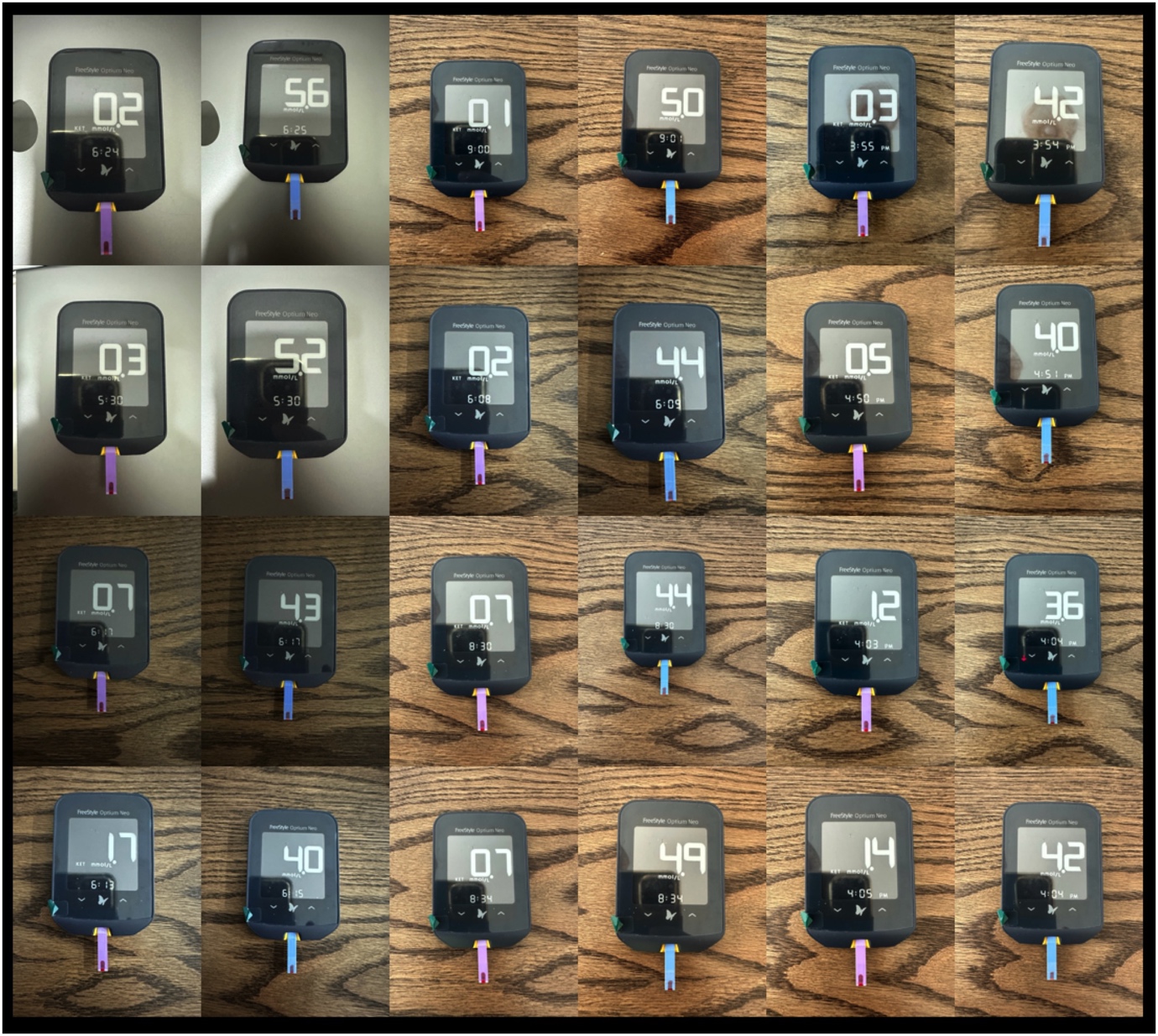


**Fig. S5.** Subject N001’s ketone and glucose levels. Photos of recorded ketone and glucose levels from OCT 30 to NOV 4, 2023.

**Fig. S6.** Measured blood pressures against 135/85 and 120/80 benchmarks. Subject N001’s blood pressures were subtracted from each respective benchmark (ΔmmHg). Positive ΔmmHg indicates lowered blood pressure and negative ΔmmHg represents measurements outside the benchmark.

**
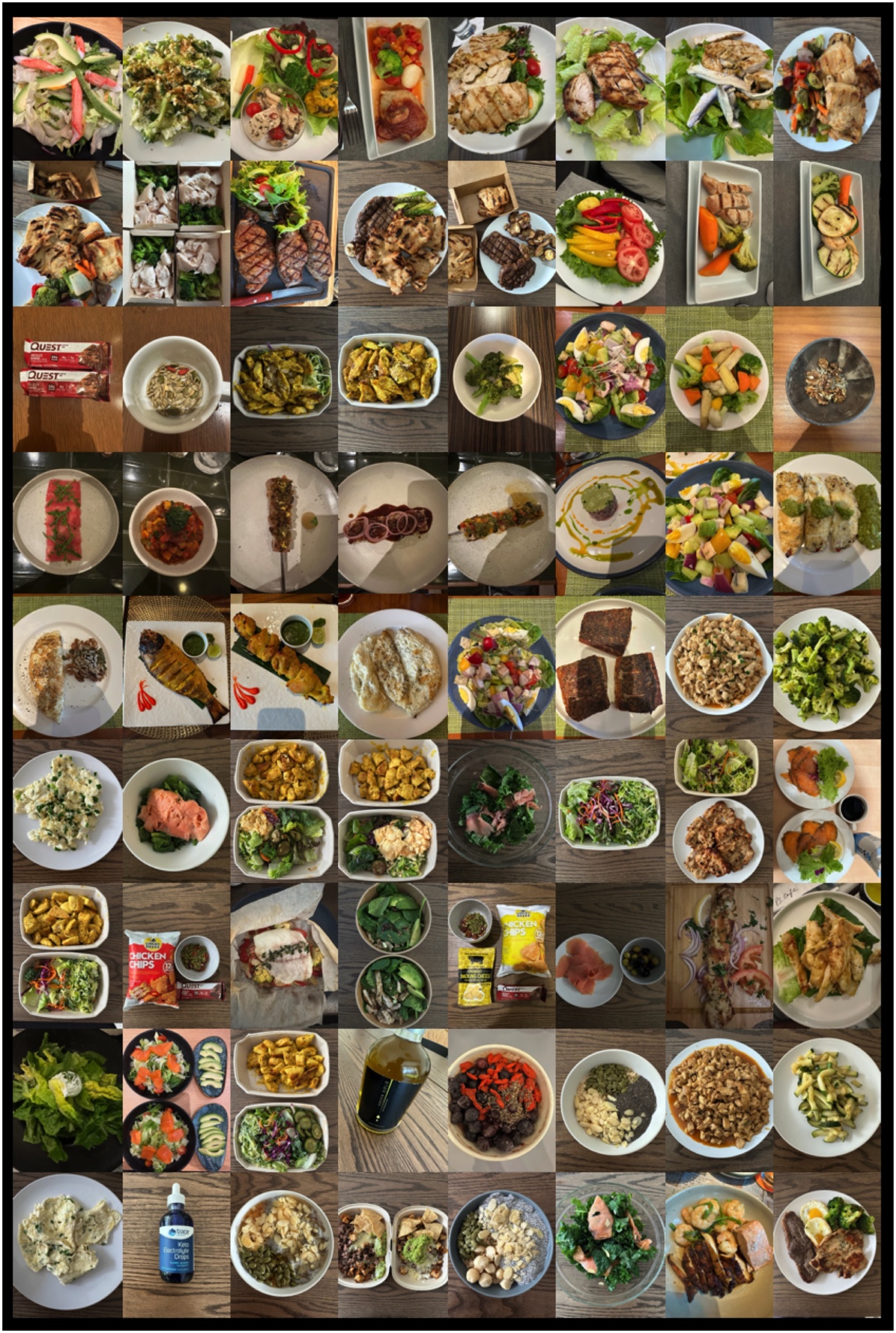
**

**Fig. S7.** Subject N001’s dietary regimen. Clean ketonic diet regimens were consistently photographed during the study.

**Table S1.** Subject N001’s blood pressure data for 72 h fast. Three blood pressure measurements were taken pre- and post-workouts (AM) as well as in the afternoon (PM) (2023) (N = 3).

| **Date** | **Diastolic (mmHg)** | | | **Systolic (mmHg)** | | |
| --- | --- | --- | --- | --- | --- | --- |
| 10.30 Pre-Workout | 118 | 122 | 124 | 79 | 72 | 75 |
| 10.30 Post-Workout | 127 | 123 | 116 | 71 | 72 | 69 |
| 10.30 Afternoon | 123 | 119 | 122 | 69 | 76 | 72 |
| 10.31 Pre-Workout | 121 | 118 | 126 | 73 | 70 | 74 |
| 10.31 Post-Workout | 123 | 127 | 123 | 73 | 74 | 76 |
| 10.31 Afternoon | 125 | 125 | 124 | 72 | 76 | 78 |
| 11.1 Pre-Workout | 126 | 119 | 122 | 76 | 71 | 77 |
| 11.1 Post-Workout | 122 | 123 | 118 | 77 | 78 | 72 |
| 11.1 Afternoon | 124 | 119 | 122 | 77 | 70 | 69 |
| 11.2 Pre-Workout | 121 | 115 | 115 | 73 | 71 | 75 |
| 11.2 Post-Workout | 120 | 117 | 116 | 70 | 68 | 64 |
| 11.2 Afternoon | 121 | 119 | 121 | 72 | 73 | 77 |

**Table S2.** Longitudinal monitoring of subject N001’s blood pressure. Three blood pressure measurements were taken at least three times a day: morning (AM; pre- and post-workouts), afternoon (PM), and evening (night time; NT) (2023) (N = 3-6).

| **Date** | **Diastolic (mmHg)** | | | **Systolic (mmHg)** | | |
| --- | --- | --- | --- | --- | --- | --- |
| 8.29 Evening | 121 | 123 | 129 | 80 | 71 | 81 |
| 8.30 Morning | 138 | 124 | 136 | 81 | 77 | 71 |
| 8.30 Afternoon | 132 | 128 | 124 | 77 | 71 | 76 |
| 8.30 Evening | 121 | 111 | 117 | 78 | 76 | 76 |
| 8.31 Morning | 134 | 129 | 117 | 83 | 73 | 76 |
| 8.31 Afternoon | 133 | 129 | 129 | 80 | 79 | 80 |
| 8.31 Evening | 124 | 121 | 123 | 81 | 74 | 85 |
| 9.1 Morning | 133 | 105 | 120 | 81 | 76 | 75 |
| 9.1 Afternoon | 127 | 104 | 121 | 80 | 81 | 76 |
| 9.1 Evening | 125 | 122 | 129 | 66 | 69 | 86 |
| 9.2 Morning | 138 | 125 | 131 | 80 | 75 | 76 |
| 9.2 Afternoon | 124 | 122 | 119 | 73 | 74 | 63 |
| 9.2 Evening | 126 | 109 | 123 | 80 | 78 | 78 |
| 9.3 Morning | 129 | 115 | 119 | 82 | 78 | 71 |
| 9.3 Afternoon | 130 | 131 | 123 | 82 | 74 | 76 |
| 9.3 Evening | 124 | 120 | 122 | 76 | 82 | 68 |
| 9.4 Morning | 105 | 125 | 118 | 74 | 76 | 70 |
| 9.4 Afternoon | 127 | 118 | 109 | 73 | 79 | 72 |
| 9.4 Evening | 127 | 118 | 120 | 74 | 72 | 74 |
| 9.5 Morning | 119 | 112 | 109 | 67 | 68 | 67 |
| 9.5 Afternoon | 121 | 123 | 129 | 80 | 71 | 81 |
| 9.5 Evening | 138 | 124 | 136 | 81 | 77 | 71 |
| 9.6 Morning | 132 | 128 | 124 | 77 | 71 | 76 |
| 9.6 Evening | 121 | 111 | 117 | 78 | 76 | 76 |
| 9.7 Morning | 134 | 129 | 117 | 83 | 73 | 76 |
| 9.7 Afternoon | 133 | 129 | 129 | 80 | 79 | 80 |
| 9.8 Morning | 124 | 121 | 123 | 81 | 74 | 85 |
| 9.8 Evening | 133 | 105 | 120 | 81 | 76 | 75 |
| 9.9 Morning | 127 | 104 | 121 | 80 | 81 | 76 |
| 9.9 Afternoon | 125 | 122 | 129 | 66 | 69 | 86 |
| 9.9 Evening | 138 | 125 | 131 | 80 | 75 | 76 |
| 9.10 Pre-Workout | 124 | 122 | 119 | 73 | 74 | 63 |
| 9.10 Post-Workout | 126 | 109 | 123 | 80 | 78 | 78 |
| 9.10 Evening | 129 | 115 | 119 | 82 | 78 | 71 |
| 9.11 Morning | 130 | 131 | 123 | 82 | 74 | 76 |
| 9.11 Afternoon | 124 | 120 | 122 | 76 | 82 | 68 |
| 9.14 Morning | 123 | 114 | 118 | 72 | 70 | 70 |
| 9.15 Afternoon | 129 | 125 | 110 | 81 | 78 | 70 |
| 9.18 Afternoon | 123 | 124 | 117 | 61 | 71 | 77 |
| 9.25 Evening | 124 | 117 | 119 | 79 | 78 | 74 |
| 9.26 Morning | 128 | 121 | 120 | 79 | 77 | 75 |
| 9.26 Afternoon | 126 | 128 | 125 | 78 | 73 | 75 |
| 9.27 Evening | 117 | 122 | 112 | 79 | 75 | 78 |
| 9.28 Pre-Workout | 121 | 119 | 117 | 77 | 77 | 80 |
| 9.28 Post-Workout | 114 | 110 | 111 | 75 | 71 | 74 |
| 9.28 Evening | 122 | 123 | 123 | 74 | 77 | 75 |
| 9.29 Morning | 117 | 117 | 120 | 76 | 76 | 70 |
| 9.29 Afternoon | 121 | 120 | 120 | 76 | 75 | 77 |
| 9.29 Evening | 121 | 116 | 115 | 75 | 71 | 73 |
| 9.30 Pre-Workout | 119 | 113 | 123 | 77 | 76 | 78 |
| 9.30 Post-Workout | 118 | 121 | 117 | 75 | 77 | 76 |
| 9.30 Afternoon | 123 | 116 | 115 | 75 | 78 | 77 |
| 10.1 Pre-Workout | 111 | 119 | 115 | 70 | 75 | 73 |
| 10.1 Post-Workout | 120 | 115 | 122 | 71 | 67 | 67 |
| 10.1 Afternoon | 120 | 118 | 116 | 70 | 75 | 77 |
| 10.1 Afternoon | 119 | 121 | 119 | 69 | 74 | 72 |
| 10.2 Pre-Workout | 120 | 112 | 119 | 75 | 78 | 76 |
| 10.2 Post-Workout | 124 | 123 | 115 | 70 | 65 | 75 |
| 10.2 Evening | 113 | 118 | 112 | 72 | 75 | 69 |
| 10.3 Pre-Workout | 116 | 119 | 120 | 77 | 76 | 76 |
| 10.3 Post-Workout | 119 | 113 | 108 | 70 | 74 | 71 |
| 10.3 Afternoon | 125 | 115 | 124 | 77 | 78 | 69 |
| 10.3 Evening | 125 | 120 | 122 | 77 | 76 | 77 |
| 10.4 Pre-Workout | 115 | 118 | 118 | 76 | 76 | 73 |
| 10.4 Post-Workout | 118 | 114 | 112 | 75 | 77 | 77 |
| 10.4 Evening | 108 | 102 | 106 | 68 | 75 | 70 |
| 10.4 Evening | 107 | 112 | 113 | 69 | 66 | 68 |
| 10.4 Evening | 114 | 118 | 109 | 75 | 65 | 75 |
| 10.5 Pre-Workout | 118 | 120 | 115 | 73 | 74 | 74 |
| 10.5 Post-Workout | 119 | 120 | 120 | 73 | 72 | 68 |
| 10.5 Evening | 114 | 117 | 113 | 70 | 70 | 62 |
| 10.6 Pre-Workout | 117 | 117 | 115 | 73 | 75 | 66 |
| 10.6 Post-Workout | 115 | 115 | 116 | 66 | 68 | 65 |
| 10.6 Evening | 120 | 112 | 117 | 76 | 69 | 73 |
| 10.7 Pre-Workout | 117 | 120 | 121 | 74 | 74 | 71 |
| 10.7 Post-Workout | 117 | 118 | 115 | 71 | 72 | 69 |
| 10.7 Evening | 113 | 111 | 110 | 77 | 73 | 66 |
| 10.8 Pre-Workout | 120 | 119 | 110 | 73 | 75 | 76 |
| 10.8 Post-Workout | 121 | 114 | 116 | 72 | 72 | 67 |
| 10.8 Morning | 122 | 112 | 111 | 75 | 74 | 75 |
| 10.10 Pre-Workout | 116 | 117 | 114 | 70 | 68 | 70 |
| 10.10 Post-Workout | 117 | 117 | 114 | 76 | 79 | 77 |
| 10.11 Pre-Workout | 118 | 116 | 113 | 78 | 73 | 76 |
| 10.11 Post-Workout | 119 | 123 | 120 | 78 | 76 | 78 |
| 10.11 Afternoon | 120 | 122 | 114 | 69 | 69 | 66 |
| 10.12 Pre-Workout | 121 | 119 | 118 | 72 | 74 | 75 |
| 10.12 Post-Workout | 118  112 | 113  107 | 112  113 | 71  69 | 74  73 | 73  73 |
| 10.12 Morning | 119 | 117 | 115 | 62 | 67 | 65 |
| 10.13 Pre-Workout | 123 | 120 | 119 | 70 | 75 | 78 |
| 10.13 Post-Workout | 122  120 | 116  113 | 120  115 | 71  75 | 73  76 | 74  76 |
| 10.13 Morning | 116 | 116 | 120 | 74 | 75 | 77 |

**Table S3.** Subject N001’s measured weights. Measurements were taken at least three time (SEP 2023) (N = 3-6).

| **Date** | **Weight (kg)** | | | | | |
| --- | --- | --- | --- | --- | --- | --- |
| 9.5 | 82.5 | 82.2 | 82.2 | 82.3 |  |  |
| 9.6 | 81.0 | 81.1 | 81.1 |  |  |  |
| 9.7 | 80.8 | 80.7 | 80.7 | 80.0 | 80.0 | 79.9 |
| 9.8 | 79.7 | 79.8 | 79.6 | 79.7 | 79.6 | 80 |
| 9.9 | 78.4 | 78.3 | 78.5 |  |  |  |
| 9.20 | 75.8 | 75.9 | 75.7 |  |  |  |
| 9.30 | 74.9 | 75.0 | 75.0 |  |  |  |
